# BRS‐Assessed Resilience, Symptom Burden, and Functional Burden in Adolescents With Depressive, Anxiety, or Somatic Symptom‐Related Disorders: A Single‐Center Real‐World Cross‐Sectional Study

**DOI:** 10.1002/brb3.71811

**Published:** 2026-10-08

**Authors:** Longhuan Zhang, Meiying Zhang, Xuejing Liu, Lingcong Wu, Yang Zhang, Jing Shan

**Affiliations:** ^1^ Department of Psychiatry and Psychology Harrison International Peace Hospital Hengshui Hebei Province China; ^2^ Department of Abdominal Surgery The Second People's Hospital of Hengshui Hengshui Hebei Province China

**Keywords:** adolescent psychiatry, Brief Resilience Scale, depressive symptoms, functional burden

## Abstract

**Background:**

Adolescents with depressive, anxiety, and somatic symptom‐related disorders often present with overlapping symptom domains and functional difficulties, but cross‐sectional associations of Brief Resilience Scale (BRS) score with multidimensional symptom and functional burden in routine outpatient care remain insufficiently characterized.

**Methods:**

This single‐center, real‐world, cross‐sectional study retrospectively analyzed routinely collected data from adolescents aged 12–18 years attending a psychological/psychiatric outpatient clinic between January 2024 and December 2025. Eligible patients had clinician‐recorded depressive, anxiety, or somatic symptom‐related disorders and completed the BRS, PHQ‐9, GAD‐7, and PHQ‐15. Associations of BRS score with symptom outcomes and predefined pragmatic functional‐burden indicators were assessed using multivariable linear, logistic, and negative binomial regression models; restricted cubic spline analyses were exploratory.

**Results:**

Among 522 screened adolescents, 447 were included. Across increasing BRS‐score quartiles, mean PHQ‐9 scores ranged from 17.1 ± 5.8 to 9.9 ± 5.2, GAD‐7 scores from 13.4 ± 5.1 to 7.2 ± 4.5, and PHQ‐15 scores from 12.2 ± 5.0 to 6.9 ± 4.3. The predefined marked functional impairment indicator ranged from 71.4% in Q1 to 30.6% in Q4 (*P* for trend <0.001). After adjustment, each 1‐SD higher BRS score was associated with lower PHQ‐9 score (*β*, −2.42; 95% CI, −3.02 to −1.82), GAD‐7 score (*β*, −1.84; 95% CI, −2.38 to −1.30), PHQ‐15 score (*β*, −1.50; 95% CI, −2.05 to −0.95), and functional impairment count (incidence rate ratio, 0.72; 95% CI, 0.64 to 0.82). Higher BRS score was also associated with lower odds of PHQ‐9 score ≥10 (OR, 0.49; 95% CI, 0.36 to 0.66), GAD‐7 score ≥10 (OR, 0.57; 95% CI, 0.43 to 0.75), PHQ‐15 score ≥10 (OR, 0.61; 95% CI, 0.45 to 0.82), and marked functional impairment (OR, 0.53; 95% CI, 0.40 to 0.71). Exploratory spline analyses showed graded inverse associations but were not interpreted as identifying clinical thresholds.

**Conclusion:**

Higher BRS score was concurrently associated with lower depressive, anxiety, and somatic symptom burden and fewer pragmatic indicators of functional burden in adolescent psychiatric outpatients. These cross‐sectional findings do not establish causality, prognostic utility, or treatment‐guiding value; longitudinal studies are needed to clarify temporal relationships and clinical utility.

## Graphical abstract

In 447 adolescents with depressive, anxiety, or somatic symptom‐related disorders, higher Brief Resilience Scale scores were consistently associated with lower depressive, anxiety, and somatic symptom burden and fewer indicators of functional impairment. These findings suggest that resilience is a clinically relevant correlate of multidimensional burden, although the cross‐sectional design does not establish causality, prognostic value, or treatment‐guiding utility.

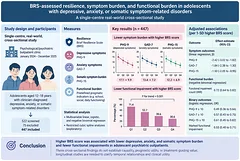

## 1 Introduction

The adolescent patients visiting the psychological or psychiatric outpatient clinics rarely present with a single and clear symptom problem (Polanczyk et al. 2015; Kieling et al. 2011; Solmi et al. 2022). In routine consultations, issues such as depressive mood, excessive worry, irritability, sleep disorders, fatigue, headaches, abdominal discomfort, avoidance of school, and family conflicts often occur simultaneously in the same visit (Mahirah et al. 2025; Lu et al. 2025). This pattern is particularly common in general hospitals, where physical discomfort may be the primary reason for seeking medical help, even though emotional distress is already quite obvious at this time (Klastrup et al. 2022; Wu et al. 2023). For clinicians, diagnostic labels are still necessary, but they often only reflect a part of the clinical situation. Adolescents recorded as having depression may also have severe anxiety and physical discomfort, obvious emotional distress, and academic dysfunction (Campo 2012; Janssens et al. 2010).

This overlap is clinically relevant because symptoms across different domains frequently co‐occur and may interact within the same clinical presentation (Bohman et al. 2012; Shanahan et al. 2015; Lallukka et al. 2019). Depressive symptoms can lead patients to reduce their enthusiasm for daily activities and participation at school, anxiety may enhance the sensitivity to physical sensations, and persistent physical discomfort may result in frequent visits to the doctor and family concerns (Bohman et al. 2012; Shanahan et al. 2015; Lee et al. 2018). In such cases, depressive, anxiety, and somatic symptoms may represent interrelated dimensions of the same clinical presentation. Therefore, the assessment of the dimensions of the burden of depression, anxiety, and physical symptoms may be more accurate in describing the severity of the disease than a simple diagnosis (Xiong et al. 2017; Hybelius et al. 2026; Leonhart et al. 2018).

Functional impairment constitutes another part of the clinical burden, which cannot be fully captured by symptom scores alone (Lee et al. 2018; Sawyer et al. 2012). Two adolescents with similar PHQ‐9 or GAD‐7 scores may differ in aspects such as whether to continue their studies, maintaining daily routines, participating in family life, and the need for frequent medical visits. In pediatric and adolescent outpatient care, functional information can complement symptom severity when characterizing clinical burden and the disruption of important developmental roles (Bohman et al. 2012; McGorry et al. 2024). Issues such as suspension of studies, social withdrawal, decline in family function, and frequent medical visits are actual manifestations of the extent to which emotional and physical symptoms disrupt important developmental roles (Klastrup et al. 2022; Bohman et al. 2012). Therefore, when studying the clinical heterogeneity of adolescent psychiatric outpatients, it is crucial to assess functional impairment along with symptom burden.

Resilience in adolescence is a broad, dynamic, and context‐dependent process rather than a fixed individual trait (Luthar et al. 2000; Stainton et al. 2019). The Brief Resilience Scale (BRS) captures a narrower self‐reported dimension of this broader construct: the perceived capacity to recover from stress. In symptomatic adolescents, a lower BRS score therefore indicates a lower self‐reported perceived capacity to recover from stress at the time of assessment. At the same time, resilience should not be regarded as a simple protective factor in cross‐sectional studies. More severe depression, anxiety, or physical discomfort itself may also reduce adolescents' confidence in their ability to recover from stress. Factors such as sleep disorders, family adversity, peer problems, and social support may also affect resilience and clinical burden. These bidirectional and contextual influences require cautious interpretation of cross‐sectional associations between BRS score and clinical burden.

Previous studies have often examined resilience in relation to depression, anxiety, or somatic symptoms separately, and many have focused on community or school populations rather than adolescents already seeking psychological or psychiatric outpatient care. However, for those adolescents who have already received treatment in psychological or psychiatric outpatient clinics, less is known about the relationship between resilience and a broader combination of symptoms. Evidence is also limited regarding how BRS score relates to pragmatic indicators of functional burden documented in outpatient records, such as school absence, repeated healthcare use, and clinician‐recorded family or social functional decline. The shape of the cross‐sectional association between BRS score and clinical burden across the observed score distribution has also been less well described.

To address these questions, we conducted a single‐center real‐world cross‐sectional study of adolescents aged 12 to 18 years attending psychological or psychiatric outpatient care with clinician‐recorded depressive, anxiety, or somatic symptom‐related disorders. Using routinely collected outpatient assessment data, we examined cross‐sectional associations of BRS score with depressive, anxiety, and somatic symptom burden and with predefined pragmatic indicators of functional burden. We also evaluated these associations after adjustment for prespecified demographic, diagnostic, clinical, and treatment‐related factors and explored their shape across the observed BRS‐score distribution.

## 2 Methods

### 2.1 Study Design and Setting

This was a single‐center, real‐world, cross‐sectional analysis of routinely collected outpatient clinical data. The study was carried out in the psychological or psychiatric outpatient clinic of Harrison International Peace Hospital. We used outpatient assessment data and electronic medical records collected during routine care from January 2024 to December 2025. The study examined cross‐sectional associations of BRS score with depressive, anxiety, and somatic symptom burden and with predefined pragmatic indicators of functional burden in adolescents with depressive, anxiety, or somatic symptom‐related disorders.

We defined the index assessment as the first outpatient visit during the study period in which the patient completed the core psychological measures and had enough clinical information in the medical record. The exposure and outcomes were measured at the same visit. Therefore, we treated the study as a cross‐sectional association study. We did not treat it as a longitudinal study or a causal analysis. Accordingly, all effect estimates were interpreted as concurrent cross‐sectional associations and not as causal, prognostic, predictive, or treatment‐guiding effects. The study followed the main principles of STROBE for observational studies. We also considered issues related to routinely collected clinical data.

### 2.2 Participants and Eligibility Criteria

Patients were eligible if they were aged 12–18 years, attended the psychological/psychiatric outpatient clinic between January 2024 and December 2025, had a clinician‐recorded diagnosis of depressive disorder, anxiety disorder, or somatic symptom‐related disorder, and completed the BRS, PHQ‐9, GAD‐7, and PHQ‐15 during routine outpatient assessment. Patients also had to have extractable demographic, diagnostic, and treatment‐related information in the electronic medical record.

Patients were excluded if they were outside the prespecified age range, had a primary diagnosis of schizophrenia‐spectrum disorder, bipolar disorder, organic mental disorder, moderate‐to‐severe intellectual disability, or severe neurodevelopmental disorder that precluded reliable self‐report. Patients requiring immediate emergency intervention or inpatient admission because of acute suicide risk were not included in the analytic sample. Suicidal ideation alone was not an exclusion criterion unless it resulted in immediate emergency referral or hospitalization before completion of the index assessment. For patients with repeated outpatient visits during the study period, only the first complete assessment was retained before eligibility screening. Of the 522 screened adolescent outpatient assessment records, 75 were excluded: outside the age range of 12–18 years (*n* = 16), primary diagnosis outside the prespecified diagnostic spectrum (*n* = 17), missing or invalid BRS, PHQ‐9, GAD‐7, or PHQ‐15 data (*n* = 27), insufficient electronic medical record data (*n* = 9), and emergency referral or immediate hospitalization before complete outpatient assessment (*n* = 6). The remaining 447 patients were included in the final analysis. No a priori sample‐size calculation was performed because this was a retrospective analysis of routinely collected clinical data; all patients meeting the eligibility criteria during the study period were included.

### 2.3 Diagnostic Assessment and Diagnostic Categories

Clinical diagnoses were assigned by outpatient psychiatrists or psychological medicine physicians as part of routine care and were coded according to ICD‐10 diagnostic categories. Depressive disorders comprised ICD‐10 F32 depressive episodes, F33 recurrent depressive disorders, and F34.1 dysthymia. Anxiety disorders comprised ICD‐10 F40 phobic anxiety disorders and F41 other anxiety disorders, including panic disorder and generalized anxiety disorder. Somatic symptom‐related disorders were used as a broad clinical term for presentations coded within the ICD‐10 F45 somatoform disorder category.

Structured diagnostic interviews were not part of routine care in this outpatient clinic, so they were not used in this study. Two trained investigators reviewed the electronic medical records to improve the consistency of diagnostic classification based on information available in routine care. They checked the chief complaint, history of present illness, clinician‐recorded diagnosis, ICD‐10 code, and treatment plan. A senior psychiatrist made the final decision when the diagnostic category was unclear. The primary diagnosis was the main reason for the index outpatient visit. Other psychiatric diagnoses were recorded as comorbidities.

### 2.4 Assessment Procedures and Psychological Measures

The BRS was used to assess the individual's self‐reported perceived capacity to recover from stress. The scale contains six items and captures this specific dimension rather than the broader dynamic construct of resilience. BRS items were scored from 1 to 5; items 2, 4, and 6 were reverse scored, and the mean of all six items was used as the total BRS score. Scores range from 1 to 5, with higher values indicating a greater self‐reported perceived capacity to recover from stress. The questionnaire versions, response options, and scoring procedures used in routine outpatient assessment were applied without modification for the present analysis. All four psychological measures were administered as Chinese‐language self‐report questionnaires during the index outpatient assessment.

Depressive symptoms were measured with the PHQ‐9, anxiety symptoms with the GAD‐7, and somatic symptom burden with the PHQ‐15 (Kroenke et al., 2010). Total scores range from 0 to 27 for PHQ‐9, 0 to 21 for GAD‐7, and 0 to 30 for PHQ‐15, with higher scores indicating greater symptom burden. PHQ‐9 score ≥10, GAD‐7 score ≥10, and PHQ‐15 score ≥10 were used to indicate at least moderate depressive, anxiety, and somatic symptom burden, respectively. Thresholds of 15 or higher were reported descriptively in Table 2 but were not included as additional binary outcomes in the main multivariable models. Internal consistency of the BRS, PHQ‐9, GAD‐7, and PHQ‐15 in the analytic cohort was assessed using Cronbach's α and reported in Table S1. These scales were used to quantify symptom severity and were not used as substitutes for clinical diagnosis.

### 2.5 Definition of Pragmatic Functional‐Burden Indicators

Functional burden was characterized using five predefined pragmatic indicators derived from symptom‐related functioning, school attendance, healthcare use, and clinician‐recorded social or family functioning available in routine outpatient records. Marked functional impairment was defined a priori as the presence of at least one of the five routinely documented indicators: patient‐reported symptom‐related functioning rated as very difficult or extremely difficult on the routine outpatient functional‐difficulty question; school absence of 4 days or more in the previous month; school suspension or interruption in the previous 6 months; 3 or more healthcare visits related to the current clinical problem in the previous 3 months; or clinician‐recorded deterioration in social or family functioning during the current episode.

A functional impairment count was calculated as the number of these five pragmatic indicators present, yielding a count from 0 to 5. Both the functional impairment count and the binary marked functional impairment variable were treated as pragmatic markers of functional burden rather than as validated psychometric functional outcomes. The definition was selected to reflect clinically meaningful impairment that could be identified in a general hospital outpatient setting without requiring additional licensed or proprietary functional assessment instruments. The operational definition, assessment window, and data source for each component are provided in Table S2.

### 2.6 Covariates and Treatment‐Related Variables

Demographic covariates included age, sex, educational stage, urban residence, only‐child status, parental marital status, and parental education. Clinical variables included primary diagnosis, psychiatric comorbidity, illness duration, first episode status, family history of mental disorders, history of self‐harm, suicidal ideation during the current episode, sleep disturbance, chronic somatic disease, and nonsevere neurological disorder. These variables were extracted from structured fields and narrative clinical notes in the electronic medical record.

Treatment‐related variables included first visit status, current psychotropic medication use, medication duration, antidepressant use, sedative or hypnotic use, and psychotherapy or counseling. For multivariable analyses, medication status was categorized as no current medication, current medication for less than 4 weeks, or current medication for 4 weeks or more. Self‐harm history and suicidal ideation were described as clinically important baseline risk characteristics but were not included in the main adjustment set because of their close conceptual overlap with current symptom severity and the potential for overadjustment in this cross‐sectional setting. An expanded sensitivity model additionally adjusted for sleep disturbance, self‐harm history, and suicidal ideation during the current episode to evaluate the robustness of the primary associations to these clinically relevant factors.

### 2.7 Exposure and Outcome Definitions

The primary exposure was the BRS score, representing the specific self‐reported capacity to recover from stress captured by the BRS. For descriptive analyses, patients were divided into quartiles of the observed BRS‐score distribution, with Q1 representing the lowest BRS scores and Q4 the highest BRS scores. For regression analyses, BRS was also analyzed as a continuous standardized variable, allowing effect estimates to be interpreted per 1‐SD higher BRS score.

The continuous symptom outcomes were PHQ‐9 score, GAD‐7 score, and PHQ‐15 score, whereas the functional impairment count was treated as a count outcome. The complementary binary outcomes were PHQ‐9 score ≥10, GAD‐7 score ≥10, PHQ‐15 score ≥10, and the predefined marked functional impairment indicator. The combined use of continuous symptom scores, a count‐based functional outcome, and binary clinical outcomes was intended to characterize complementary dimensions of clinical burden.

### 2.8 Data Extraction, Missing Data, and Quality Control

Data came from electronic medical records and routine outpatient psychological assessment forms. The research team used a standard data collection template. The extracted data included demographic information, diagnostic codes, clinician‐recorded diagnoses, illness course, treatment status, risk history, somatic comorbidity, and scores from the main psychological measures. The investigators checked the records for internal consistency. When an entry was unclear, the investigators discussed it and reached a final decision.

Patients were excluded from the final analysis if they had missing core exposure or outcome data. These data included BRS, PHQ‐9, GAD‐7, and PHQ‐15 scores. Questionnaires were treated as invalid if they had many missing items, clear patterned responses, or inconsistent answers that made scoring unreliable. The analytic cohort therefore comprised complete cases for the core BRS exposure and symptom outcomes. No multiple imputation was performed because records with missing or invalid core questionnaire data were excluded before construction of the analytic cohort. All covariates included in the multivariable models were available for the 447 participants in the final analytic cohort; therefore, no covariate imputation was performed. For patients with more than one outpatient visit during the study period, the analysis used only the first complete outpatient assessment. This approach helped keep the observations independent.

### 2.9 Statistical Analysis

Continuous variables were summarized as mean ± SD or median with interquartile range, depending on distributional characteristics. Categorical variables were summarized as *n* (%). Baseline characteristics were summarized descriptively across BRS‐score quartiles; continuous variables were compared using one‐way analysis of variance or the Kruskal–Wallis test, as appropriate, and categorical variables using Pearson χ^2^ tests. Baseline *P* values were unadjusted and descriptive only and were not used to select covariates or to test the primary study hypothesis. For descriptive trend analyses, BRS‐score quartile rank (1–4) was entered as an ordinal predictor in linear regression for continuous symptom scores, logistic regression for binary outcomes, and negative binomial regression for the functional impairment count.

Multivariable linear regression was used for continuous symptom outcomes, multivariable logistic regression was used for binary clinical outcomes, and negative binomial regression was used for the functional impairment count. Poisson dispersion was assessed before fitting the count outcome model, and negative binomial regression was selected for the functional impairment count because the Poisson model showed overdispersion. Models were adjusted for age, sex, educational stage, parental marital status, parental education, primary diagnosis, psychiatric comorbidity, illness duration, first episode, medication status, psychotherapy or counseling, and chronic somatic disease. Covariates were prespecified on the basis of clinical relevance and availability in routine outpatient records rather than selected according to univariable *P* values. The functional impairment count was analyzed separately using negative binomial regression and is reported in Table 3. Restricted cubic spline models were fitted as exploratory shape analyses using continuous BRS score, with three knots at the 10th, 50th, and 90th percentiles and BRS = 2.8 as the reference value. Overall association was assessed jointly across the BRS spline terms, and evidence of nonlinearity was evaluated from the nonlinear spline components. The spline analyses were not used to derive screening, prognostic, treatment‐selection, or other clinical decision thresholds. Sensitivity analyses were conducted by excluding patients who had received psychotropic medication for less than 4 weeks and by additionally adjusting the primary models for sleep disturbance, self‐harm history, and suicidal ideation during the current episode. Regression diagnostics included assessment of multicollinearity using variance inflation factors, inspection of residual distributions and residual‐versus‐fitted plots for linear models, assessment of influential observations using Cook's distance, and dispersion assessment for count models. All analyses were performed using R version 4.6.0; linear and logistic regression models were fitted using the stats package, negative binomial models using the MASS package, restricted cubic spline analyses using the rms package, and multicollinearity diagnostics using the car package. All tests were two sided, and *P* < 0.05 was considered statistically significant.

### 2.10 Ethics Approval and Patient Safety

The study protocol was reviewed and approved by the Institutional Ethics Committee of Harrison International Peace Hospital (approval number: 2024238). This study used routinely collected outpatient assessment data and electronic medical record information. The requirement for written informed consent was waived by the ethics committee in accordance with the current institutional regulations. Direct personal identifiers were removed before analysis, and the analytic dataset contained only study‐specific identifiers. The study was conducted in accordance with the Declaration of Helsinki.

The study population consisted of adolescents attending the psychological and psychiatric outpatient clinic of Harrison International Peace Hospital. Patient safety was managed by the clinical team as part of routine care, rather than as part of the research analysis. Patients with self‐harm risk, suicidal ideation, or other urgent safety concerns received routine risk assessment and crisis intervention services when clinically indicated. The research data did not influence clinical decision‐making, treatment selection, referral arrangements, or emergency care.

## 3 Results

### 3.1 Study Population and Baseline Characteristics Across BRS‐Score Quartiles

Between January 2024 and December 2025, 522 adolescent outpatients were retrospectively screened at the psychological/psychiatric outpatient clinic (Figure 1). After applying the predefined eligibility criteria, 447 patients aged 12–18 years were included in the final analysis and categorized according to BRS‐score quartiles: Q1, lowest BRS score (*n* = 112); Q2 (*n* = 112); Q3 (*n* = 112); and Q4, highest BRS score (*n* = 111). The mean BRS score was 2.8 ± 0.8 in the overall cohort and increased stepwise from 1.8 ± 0.3 in Q1 to 3.8 ± 0.4 in Q4. The mean age was 15.1 ± 1.6 years, and 281 patients (62.9%) were female. Age, sex, educational stage, urban residence, only‐child status, and parental educational level were broadly comparable across BRS‐score quartiles. The proportion of patients with divorced or separated parents was numerically lower across increasing BRS‐score quartiles, ranging from 28.6% in Q1 to 14.4% in Q4. Primary psychiatric diagnoses were broadly comparable across groups: depressive disorders accounted for 50.1% of the cohort, anxiety disorders for 34.2%, and somatic symptom‐related disorders for 15.7% (Table 1).

**FIGURE 1:**
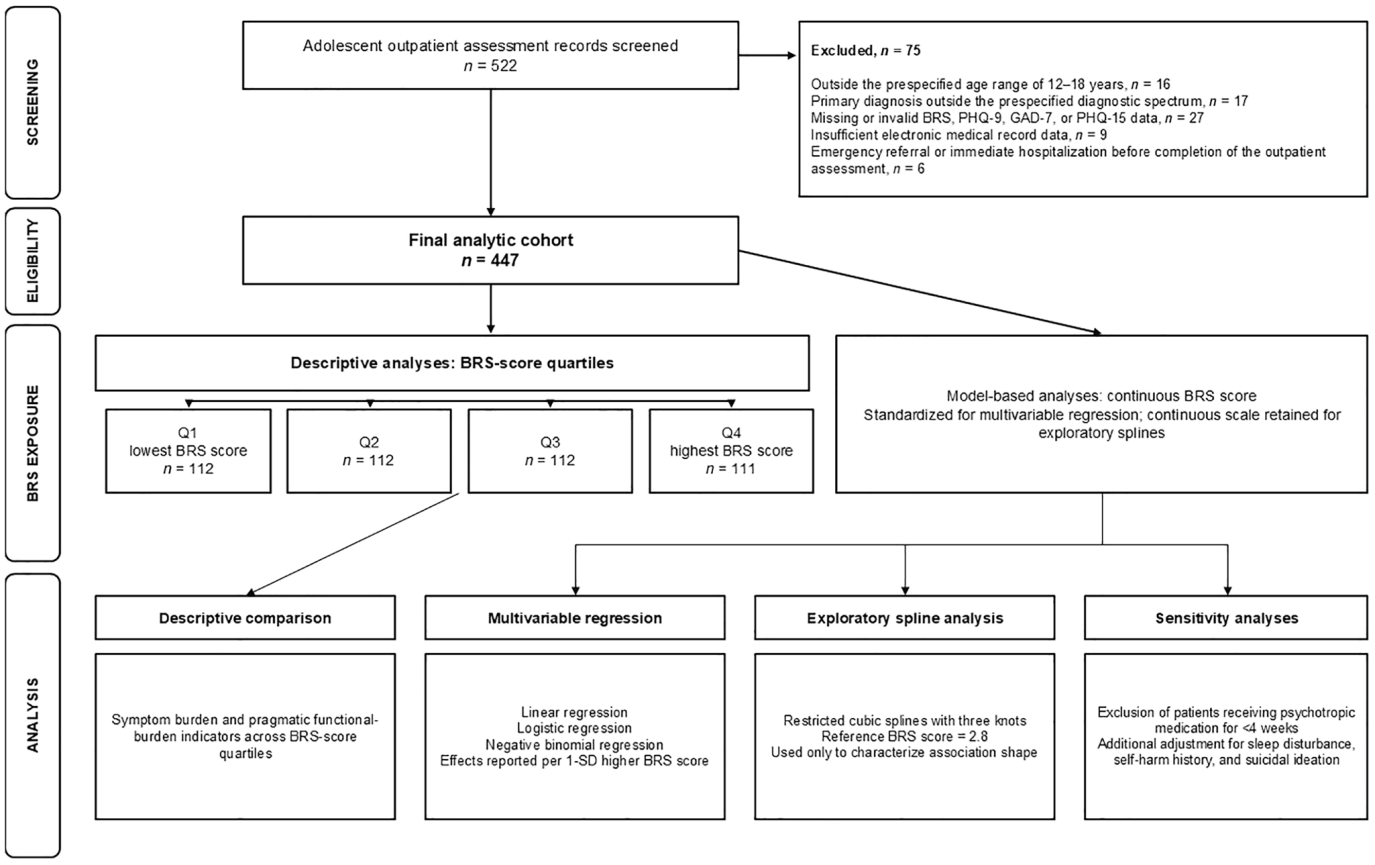
Flow diagram of cohort selection and analytic framework. A total of 522 adolescent outpatient assessment records were screened. Seventy‐five were excluded because of age outside the prespecified range (*n* = 16), a primary diagnosis outside the prespecified diagnostic spectrum (*n* = 17), missing or invalid BRS, PHQ‐9, GAD‐7, or PHQ‐15 data (*n* = 27), insufficient electronic medical record data (*n* = 9), or emergency referral or immediate hospitalization before completion of the outpatient assessment (*n* = 6). The final analytic cohort comprised 447 adolescents. For descriptive analyses, participants were classified into BRS‐score quartiles: Q1 (*n* = 112), Q2 (*n* = 112), Q3 (*n* = 112), and Q4 (*n* = 111). Continuous standardized BRS score was used in the multivariable regression models, and continuous BRS score was used in exploratory restricted cubic spline analyses.

**TABLE 1:** Baseline characteristics of adolescent outpatients across quartiles of the BRS score.

Characteristic	Overall (*n* = 447)	Q1: lowest BRS score (*n* = 112)	Q2 (*n* = 112)	Q3 (*n* = 112)	Q4: highest BRS score (*n* = 111)	Descriptive *P* value
BRS score	2.8 ± 0.8	1.8 ± 0.3	2.5 ± 0.2	3.1 ± 0.2	3.8 ± 0.4	<0.001
Age, years	15.1 ± 1.6	15.4 ± 1.5	15.1 ± 1.6	15.0 ± 1.7	14.8 ± 1.7	0.071
Female sex, *n* (%)	281 (62.9)	78 (69.6)	74 (66.1)	68 (60.7)	61 (55.0)	0.117
Senior high school or vocational school, *n* (%)	239 (53.5)	67 (59.8)	61 (54.5)	58 (51.8)	53 (47.7)	0.327
Urban residence, *n* (%)	279 (62.4)	69 (61.6)	72 (64.3)	71 (63.4)	67 (60.4)	0.931
Only child, *n* (%)	207 (46.3)	47 (42.0)	52 (46.4)	54 (48.2)	54 (48.6)	0.739
Divorced or separated parents, *n* (%)	101 (22.6)	32 (28.6)	27 (24.1)	26 (23.2)	16 (14.4)	0.082
At least one parent with college education or above, *n* (%)	132 (29.5)	26 (23.2)	30 (26.8)	35 (31.3)	41 (36.9)	0.131
Primary diagnosis, *n* (%)						0.784
Depressive disorders	224 (50.1)	62 (55.4)	58 (51.8)	54 (48.2)	50 (45.0)	
Anxiety disorders	153 (34.2)	34 (30.4)	36 (32.1)	39 (34.8)	44 (39.6)	
Somatic symptom‐related disorders	70 (15.7)	16 (14.3)	18 (16.1)	19 (17.0)	17 (15.3)	
Any psychiatric comorbidity, *n* (%)	215 (48.1)	66 (58.9)	55 (49.1)	50 (44.6)	44 (39.6)	0.029
Illness duration, months	8.0 (3.0–18.0)	10.0 (4.0–24.0)	8.0 (3.0–18.0)	7.0 (3.0–16.0)	6.0 (2.0–14.0)	0.028
First episode, *n* (%)	267 (59.7)	58 (51.8)	63 (56.2)	70 (62.5)	76 (68.5)	0.061
Family history of mental disorders, *n* (%)	67 (15.0)	20 (17.9)	18 (16.1)	15 (13.4)	14 (12.6)	0.673
History of self‐harm, *n* (%)	96 (21.5)	38 (33.9)	26 (23.2)	18 (16.1)	14 (12.6)	<0.001
Suicidal ideation in current episode, *n* (%)	123 (27.5)	44 (39.3)	34 (30.4)	26 (23.2)	19 (17.1)	0.002
Sleep disturbance recorded, *n* (%)	286 (64.0)	81 (72.3)	76 (67.9)	69 (61.6)	60 (54.1)	0.028
Chronic somatic disease, *n* (%)	54 (12.1)	15 (13.4)	14 (12.5)	13 (11.6)	12 (10.8)	0.942
Nonsevere neurological disorder, *n* (%)	22 (4.9)	6 (5.4)	6 (5.4)	5 (4.5)	5 (4.5)	0.980
First visit to the study clinic, *n* (%)	251 (56.2)	60 (53.6)	63 (56.2)	64 (57.1)	64 (57.7)	0.930
Current psychotropic medication, *n* (%)	201 (45.0)	58 (51.8)	52 (46.4)	47 (42.0)	44 (39.6)	0.275
Psychotropic medication for ≥4 weeks, *n* (%)	151 (33.8)	42 (37.5)	39 (34.8)	36 (32.1)	34 (30.6)	0.712
Antidepressant use, *n* (%)	174 (38.9)	49 (43.8)	45 (40.2)	41 (36.6)	39 (35.1)	0.553
Sedative or hypnotic use, *n* (%)	51 (11.4)	16 (14.3)	14 (12.5)	12 (10.7)	9 (8.1)	0.513
Psychotherapy or counseling, *n* (%)	116 (26.0)	25 (22.3)	27 (24.1)	30 (26.8)	34 (30.6)	0.518

*Note*: Values are mean ± SD, median (IQR), or *n* (%). Percentages may not total 100 because of rounding. The BRS score reflects the specific perceived capacity to recover from stress captured by the Brief Resilience Scale and should not be interpreted as a comprehensive measure of the broader resilience construct. *P* values are unadjusted and descriptive only; categorical *P* values were calculated using Pearson χ^2^ tests, whereas continuous‐variable comparisons follow the prespecified distribution‐appropriate tests. *P* values were not used to select covariates for multivariable models.

Abbreviations: BRS = Brief Resilience Scale, IQR = interquartile range.

Several clinical risk characteristics showed descriptive gradients across BRS‐score quartiles. Any psychiatric comorbidity was present in 215 patients (48.1%) and was more frequent in Q1 than in Q4 (58.9% vs. 39.6%). Illness duration also differed across groups, with a longer median duration in Q1 than in Q4 (10.0 months [IQR, 4.0–24.0] vs. 6.0 months [IQR, 2.0–14.0]). The proportion of first‐episode patients showed a numerical gradient from 51.8% in Q1 to 68.5% in Q4. Self‐harm history and suicidal ideation during the current episode showed inverse descriptive gradients across BRS‐score quartiles: self‐harm history ranged from 33.9% in Q1 to 12.6% in Q4, while suicidal ideation ranged from 39.3% to 17.1%. Sleep disturbance was also more frequently recorded in lower BRS‐score quartiles, ranging from 72.3% in Q1 to 54.1% in Q4. In contrast, chronic somatic disease, nonsevere neurological disorder, first visit status, current psychotropic medication use, medication duration of at least 4 weeks, antidepressant use, sedative or hypnotic use, and psychotherapy or counseling showed no clear differences across BRS‐score quartiles. All baseline *P* values in Table 1 are descriptive and were not used for covariate selection.

### 3.2 Symptom Burden and Pragmatic Functional‐Burden Indicators Across BRS‐Score Quartiles

Symptom burden showed a graded inverse cross‐sectional pattern across BRS‐score quartiles. The mean PHQ‐9 score was 13.6 ± 6.1 in the overall cohort and ranged from 17.1 ± 5.8 in Q1 to 9.9 ± 5.2 in Q4 (*P* for trend <0.001). A similar pattern was observed for anxiety and somatic symptoms: mean GAD‐7 scores declined from 13.4 ± 5.1 in Q1 to 7.2 ± 4.5 in Q4, and mean PHQ‐15 scores declined from 12.2 ± 5.0 to 6.9 ± 4.3 across the same quartiles (both *P* for trend <0.001). The prevalence of at least moderate depressive symptoms, defined as PHQ‐9 score ≥10, was 58.2% overall and ranged from 82.1% in Q1 to 34.2% in Q4. More severe depressive symptoms, defined as PHQ‐9 score ≥15, were also less frequent across increasing BRS‐score quartiles, ranging from 62.5% in Q1 to 16.2% in Q4. Comparable gradients were observed for anxiety and somatic symptom burden: GAD‐7 score ≥10 decreased from 67.9% to 25.2%, GAD‐7 score ≥15 from 37.5% to 9.9%, PHQ‐15 score ≥10 from 58.0% to 19.8%, and PHQ‐15 score ≥15 from 28.6% to 7.2% across Q1 to Q4, respectively (all *P* for trend <0.001) (Table 2; Figure 2A–C).

**TABLE 2:** Symptom burden and pragmatic functional‐burden indicators across quartiles of the BRS score.

Characteristic	Overall (*n* = 447)	Q1: lowest BRS score (*n* = 112)	Q2 (*n* = 112)	Q3 (*n* = 112)	Q4: highest BRS score (*n* = 111)	*P* for trend
PHQ‐9 score	13.6 ± 6.1	17.1 ± 5.8	14.8 ± 5.6	12.4 ± 5.4	9.9 ± 5.2	<0.001
PHQ‐9 score ≥10, *n* (%)	260 (58.2)	92 (82.1)	73 (65.2)	57 (50.9)	38 (34.2)	<0.001
PHQ‐9 score ≥15, *n* (%)	166 (37.1)	70 (62.5)	48 (42.9)	30 (26.8)	18 (16.2)	<0.001
GAD‐7 score	10.3 ± 5.4	13.4 ± 5.1	11.1 ± 5.0	9.3 ± 4.8	7.2 ± 4.5	<0.001
GAD‐7 score ≥10, *n* (%)	202 (45.2)	76 (67.9)	55 (49.1)	43 (38.4)	28 (25.2)	<0.001
GAD‐7 score ≥15, *n* (%)	99 (22.1)	42 (37.5)	28 (25.0)	18 (16.1)	11 (9.9)	<0.001
PHQ‐15 score	9.5 ± 5.2	12.2 ± 5.0	10.1 ± 4.8	8.7 ± 4.6	6.9 ± 4.3	<0.001
PHQ‐15 score ≥10, *n* (%)	172 (38.5)	65 (58.0)	48 (42.9)	37 (33.0)	22 (19.8)	<0.001
PHQ‐15 score ≥15, *n* (%)	77 (17.2)	32 (28.6)	22 (19.6)	15 (13.4)	8 (7.2)	<0.001
Very or extremely difficult symptom‐related functioning, *n* (%)	174 (38.9)	65 (58.0)	50 (44.6)	35 (31.3)	24 (21.6)	<0.001
School absence ≥4 days in the past month, *n* (%)	117 (26.2)	41 (36.6)	33 (29.5)	26 (23.2)	17 (15.3)	<0.001
School suspension or interruption in the past 6 months, *n* (%)	59 (13.2)	22 (19.6)	17 (15.2)	12 (10.7)	8 (7.2)	0.004
Repeated healthcare visits ≥3 in the past 3 months, *n* (%)	101 (22.6)	35 (31.3)	29 (25.9)	22 (19.6)	15 (13.5)	0.001
Clinician‐recorded social or family functional decline, *n* (%)	150 (33.6)	56 (50.0)	42 (37.5)	30 (26.8)	22 (19.8)	<0.001
Marked functional impairment, *n* (%)	227 (50.8)	80 (71.4)	64 (57.1)	49 (43.8)	34 (30.6)	<0.001
Functional impairment count	1.0 (0.0–2.0)	2.0 (1.0–3.0)	1.0 (0.0–2.0)	0.0 (0.0–2.0)	0.0 (0.0–1.0)	<0.001

*Note*: Values are mean ± SD, median (IQR), or *n* (%). PHQ‐9 score ≥10, GAD‐7 score ≥10, and PHQ‐15 score ≥10 are the complementary binary outcomes used in multivariable models; thresholds ≥15 are descriptive only. Marked functional impairment was defined a priori as the presence of at least one of the five routinely documented indicators. The functional impairment count is the number of indicators present (range 0–5). Both variables are pragmatic markers of functional burden derived from routine clinical records and do not constitute a formally validated functional scale. *P* values for trend were calculated using BRS quartile rank as an ordinal predictor.

Abbreviations: BRS = Brief Resilience Scale, GAD‐7 = 7‐item Generalized Anxiety Disorder scale, IQR = interquartile range, PHQ‐9 = 9‐item Patient Health Questionnaire, PHQ‐15 = 15‐item Patient Health Questionnaire.

**FIGURE 2:**
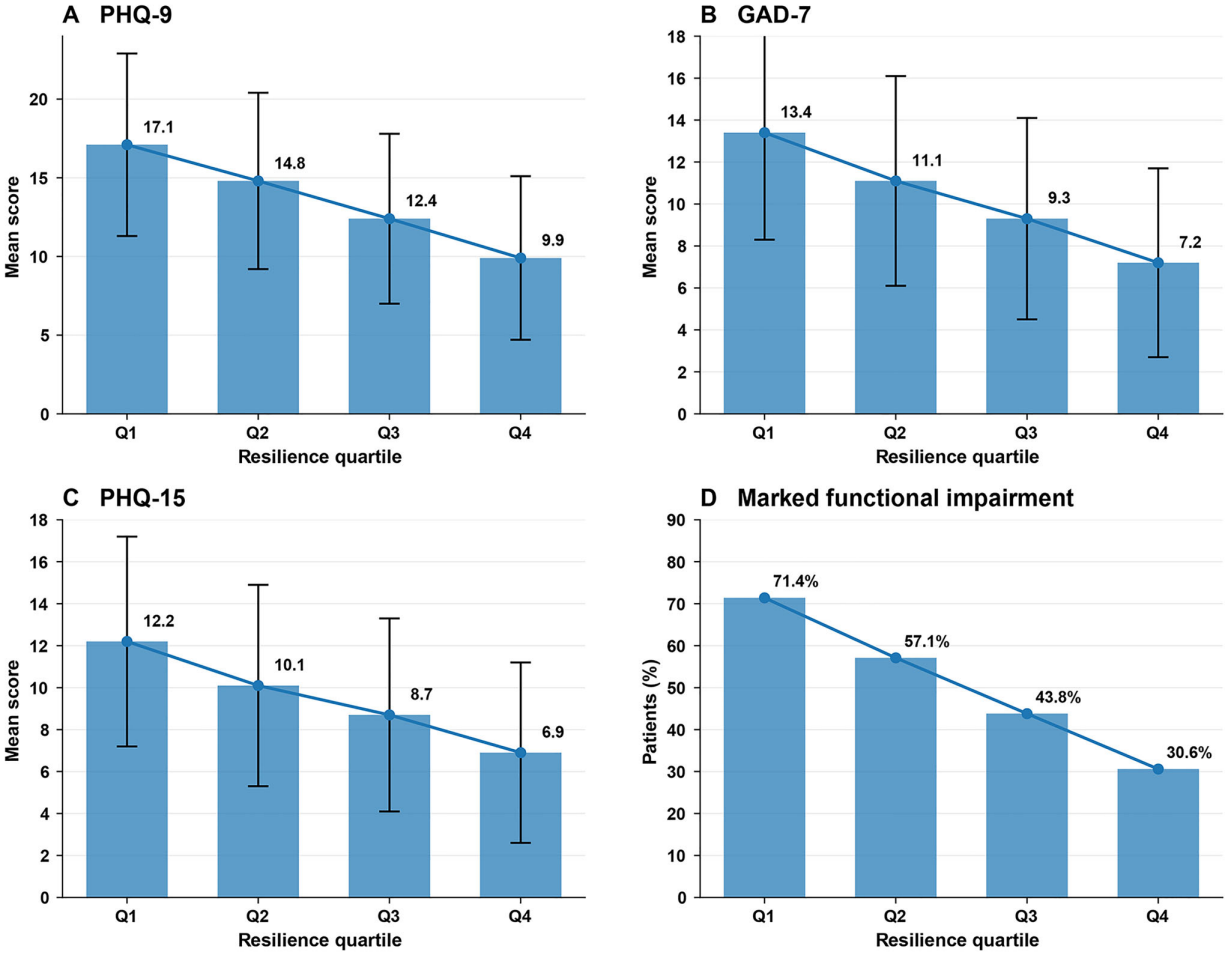
Symptom burden and pragmatic functional burden across BRS‐score quartiles. Mean PHQ‐9 (A), GAD‐7 (B), and PHQ‐15 (C) scores and the proportion of patients with marked functional impairment (D) are shown across BRS‐score quartiles. Bars in panels A–C represent means and error bars represent SDs. Marked functional impairment was defined as the presence of at least one of the five predefined pragmatic functional‐burden indicators derived from routine clinical records and does not represent a validated functional scale. *P* values for trend were calculated using BRS‐score quartile rank as an ordinal predictor. These analyses are descriptive cross‐sectional comparisons and should not be interpreted as causal effects.

The predefined pragmatic functional‐burden indicators showed similar inverse patterns across BRS‐score quartiles. Very or extremely difficult symptom‐related functioning was reported in 174 patients (38.9%) overall, with a stepwise decrease from 58.0% in Q1 to 21.6% in Q4 (*P* for trend <0.001). School absence of at least 4 days in the past month declined from 36.6% to 15.3%, school suspension or interruption in the past 6 months from 19.6% to 7.2%, repeated healthcare visits from 31.3% to 13.5%, and clinician‐recorded social or family functional decline from 50.0% to 19.8% across Q1 to Q4. The predefined marked functional impairment indicator was present in 227 patients (50.8%) overall, ranging from 71.4% in Q1 to 30.6% in Q4 (*P* for trend <0.001). The functional impairment count showed a similar pattern, with median values of 2.0, 1.0, 0.0, and 0.0 across Q1 to Q4, respectively (*P* for trend <0.001) (Table 2; Figure 2D).

### 3.3 Higher BRS Score Was Associated With Lower Symptom and Pragmatic Functional Burden After Multivariable Adjustment

In multivariable‐adjusted models, higher BRS score was associated with lower symptom scores, a lower count of pragmatic functional‐burden indicators, and lower odds of the complementary binary symptom‐burden outcomes and marked functional impairment. Each 1‐SD higher BRS score was associated with a 2.42‐point lower PHQ‐9 score (adjusted *β*, −2.42; 95% CI, −3.02 to −1.82; *P* < 0.001), a 1.84‐point lower GAD‐7 score (adjusted *β*, −1.84; 95% CI, −2.38 to −1.30; *P* < 0.001), and a 1.50‐point lower PHQ‐15 score (adjusted *β*, −1.50; 95% CI, −2.05 to −0.95; *P* < 0.001). For the functional impairment count, each 1‐SD higher BRS score was associated with a lower expected count of impairment indicators in the negative binomial model (adjusted IRR, 0.72; 95% CI, 0.64 to 0.82; *P* < 0.001). The adjusted associations with PHQ‐9, GAD‐7, and PHQ‐15 scores are shown in Figure 3A, whereas the negative binomial estimate for the functional impairment count is reported in Table 3; all models used the same prespecified adjustment set.

**FIGURE 3:**
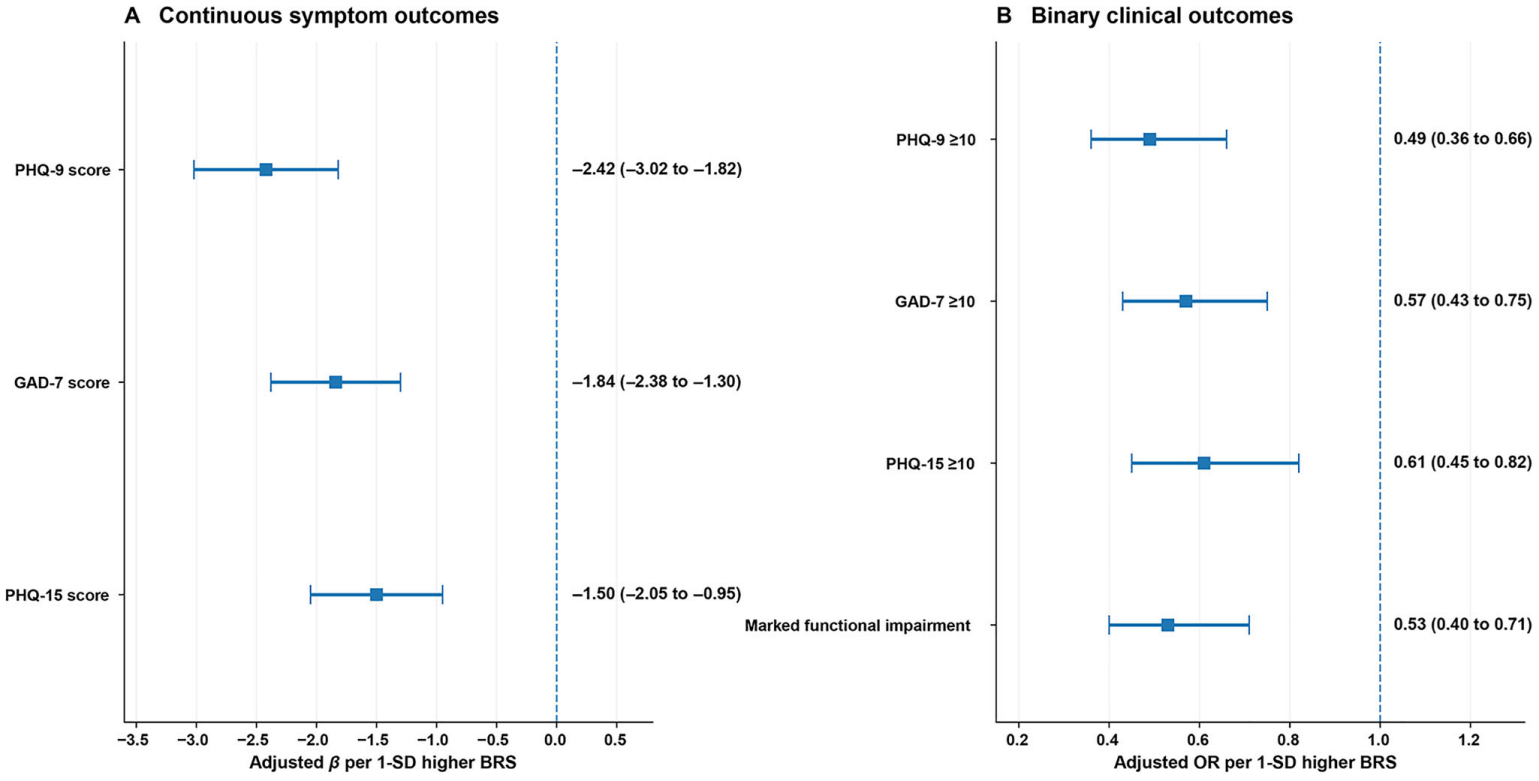
Adjusted cross‐sectional associations of BRS score with symptom and functional burden. Adjusted cross‐sectional associations per 1‐SD higher BRS score are shown for continuous symptom outcomes (A) and complementary binary clinical outcomes (B). Squares indicate adjusted estimates and horizontal lines indicate 95% CIs. Panel A shows adjusted *β* coefficients for PHQ‐9, GAD‐7, and PHQ‐15 scores. Panel B shows adjusted ORs for PHQ‐9 score ≥10, GAD‐7 score ≥10, PHQ‐15 score ≥10, and marked functional impairment. Marked functional impairment is a predefined pragmatic marker of functional burden rather than a validated functional scale. Models were adjusted for age, sex, educational stage, parental marital status, parental education, primary diagnosis, psychiatric comorbidity, illness duration, first episode, medication status, psychotherapy or counseling, and chronic somatic disease. The functional impairment count was analyzed separately using negative binomial regression and is reported in Table 3. Estimates represent concurrent associations and do not indicate causal, prognostic, predictive, or treatment‐guiding effects.

**TABLE 3:** Multivariable‐adjusted cross‐sectional associations of BRS score with symptom burden and pragmatic functional‐burden indicators.

Outcome	Outcome definition	Model	Adjusted estimate per 1‐SD higher BRS	95% CI	*P* value
**Continuous symptom outcomes**					
PHQ‐9 score	Total score, range 0–27	Linear regression	*β* = −2.42	−3.02 to −1.82	<0.001
GAD‐7 score	Total score, range 0–21	Linear regression	*β* = −1.84	−2.38 to −1.30	<0.001
PHQ‐15 score	Total score, range 0–30	Linear regression	*β* = −1.50	−2.05 to −0.95	<0.001
**Pragmatic functional‐burden count outcome**					
Functional impairment count	Count of five pragmatic indicators, range 0–5	Negative binomial regression	IRR = 0.72	0.64 to 0.82	<0.001
**Complementary binary clinical outcomes**					
Moderate or greater depressive symptoms	PHQ‐9 score ≥10	Logistic regression	OR = 0.49	0.36 to 0.66	<0.001
Moderate or greater anxiety symptoms	GAD‐7 score ≥10	Logistic regression	OR = 0.57	0.43 to 0.75	<0.001
Moderate or greater somatic symptom burden	PHQ‐15 score ≥10	Logistic regression	OR = 0.61	0.45 to 0.82	0.001
Marked functional impairment	≥1 of five predefined pragmatic functional‐burden indicators	Logistic regression	OR = 0.53	0.40 to 0.71	<0.001

*Note*: BRS was standardized before model fitting. Estimates represent cross‐sectional associations per 1‐SD higher BRS score and should not be interpreted as causal, prognostic, or treatment effects. All models were adjusted for age, sex, educational stage, parental marital status, parental education, primary diagnosis, psychiatric comorbidity, illness duration, first episode, medication status, psychotherapy or counseling, and chronic somatic disease. Medication status was categorized as no current medication, current medication for <4 weeks, or current medication for ≥4 weeks. Functional impairment variables are pragmatic markers of functional burden rather than validated psychometric outcomes.

Abbreviations: BRS = Brief Resilience Scale, CI = confidence interval, GAD‐7 = 7‐item Generalized Anxiety Disorder scale, IRR = incidence rate ratio, OR = odds ratio, PHQ‐9 = 9‐item Patient Health Questionnaire, PHQ‐15 = 15‐item Patient Health Questionnaire.

In logistic regression models, higher BRS score was also associated with lower odds of the complementary binary symptom‐burden outcomes and marked functional impairment. Each 1‐SD higher BRS score was associated with lower odds of moderate or greater depressive symptoms (PHQ‐9 score ≥10: adjusted OR, 0.49; 95% CI, 0.36 to 0.66; *P* < 0.001), moderate or greater anxiety symptoms (GAD‐7 score ≥10: adjusted OR, 0.57; 95% CI, 0.43 to 0.75; *P* < 0.001), and moderate or greater somatic symptom burden (PHQ‐15 score ≥10: adjusted OR, 0.61; 95% CI, 0.45 to 0.82; *P* = 0.001). The association was also evident for marked functional impairment, with each 1‐SD higher BRS score corresponding to lower odds of impairment (adjusted OR, 0.53; 95% CI, 0.40 to 0.71; *P* < 0.001). The adjusted estimates were directionally consistent with the descriptive patterns across BRS‐score quartiles; robustness to alternative analytic specifications was evaluated separately in the sensitivity analyses (Table 3; Figure 3B).

### 3.4 Exploratory Spline Analyses and Sensitivity Analyses

Exploratory restricted cubic spline analyses showed graded inverse cross‐sectional associations between BRS score and the clinical outcomes after multivariable adjustment. Using a BRS score of 2.8 as the reference, a lower BRS score of 2.0 was associated with higher adjusted symptom burden across the three continuous outcomes: PHQ‐9 score was higher by 2.90 points (95% CI, 1.80 to 4.00), GAD‐7 score by 2.10 points (95% CI, 1.20 to 3.10), and PHQ‐15 score by 1.70 points (95% CI, 0.80 to 2.70). Conversely, a higher BRS score of 4.0 was associated with lower adjusted symptom burden: PHQ‐9 score was lower by 3.40 points (95% CI, −4.60 to −2.20), GAD‐7 score by 3.00 points (95% CI, −4.10 to −1.90), and PHQ‐15 score by 2.50 points (95% CI, −3.60 to −1.40). The overall associations were significant for all three symptom outcomes (all *P* for overall association <0.001). Evidence of nonlinearity was modest for PHQ‐9 score (*P* for nonlinearity = 0.038), whereas the associations with GAD‐7 and PHQ‐15 scores were closer to linear (*P* for nonlinearity = 0.086 and 0.112, respectively). For marked functional impairment, the spline model likewise showed modest evidence of nonlinearity (*P* for nonlinearity = 0.044) (Table S4; Figure 4). These spline analyses were used to describe the shape of the observed associations and were not interpreted as identifying clinical thresholds, screening cutoffs, prognostic cutoffs, or treatment decision points.

**FIGURE 4:**
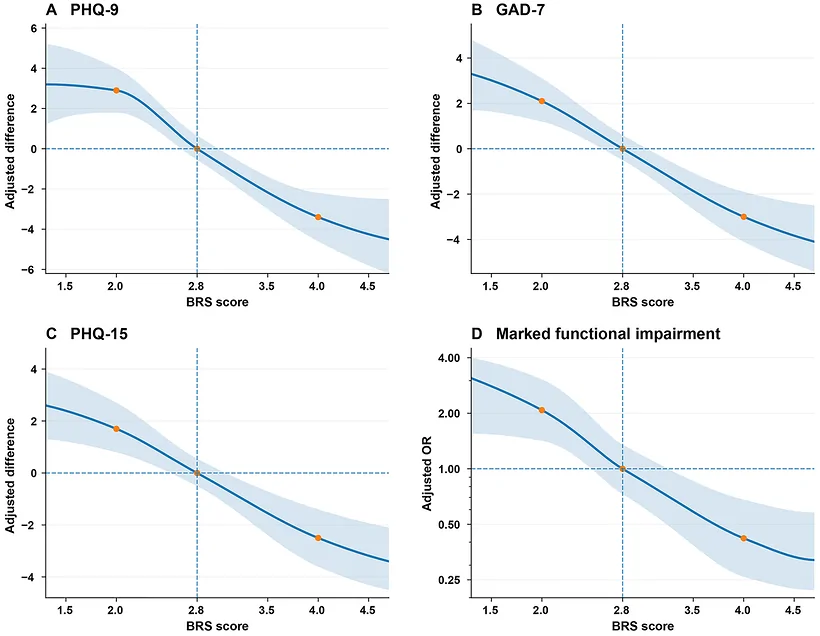
Exploratory restricted cubic spline associations of BRS score with clinical burden. Restricted cubic spline curves show exploratory adjusted cross‐sectional associations of BRS score with PHQ‐9 score (A), GAD‐7 score (B), PHQ‐15 score (C), and marked functional impairment (D). Solid lines indicate adjusted estimates and shaded areas indicate 95% CIs. The vertical dashed line denotes the reference BRS score of 2.8. Models used the same prespecified adjustment set as the main analyses. Marked functional impairment represents a pragmatic functional‐burden marker rather than a validated functional scale. These spline analyses were conducted solely to characterize the shape of the observed associations and were not used to derive clinical thresholds, screening cutoffs, prognostic cutoffs, or treatment decision points.

Two sensitivity analyses were conducted to evaluate the robustness of the primary associations. First, after excluding 50 patients who had received psychotropic medication for less than 4 weeks, 397 patients remained for analysis. The estimated associations remained directionally consistent with the main models in the restricted cohort. In this restricted cohort, each 1‐SD higher BRS score remained associated with lower PHQ‐9 score (adjusted *β*, −2.31; 95% CI, −2.96 to −1.66; *P* < 0.001), lower GAD‐7 score (adjusted *β*, −1.76; 95% CI, −2.34 to −1.18; *P* < 0.001), lower PHQ‐15 score (adjusted *β*, −1.43; 95% CI, −2.01 to −0.85; *P* < 0.001), and lower functional impairment count (adjusted IRR, 0.74; 95% CI, 0.65 to 0.84; *P* < 0.001). The corresponding binary‐outcome estimates were also directionally consistent with the main models and are reported in Table 4. Second, in the full analytic cohort, additional adjustment for sleep disturbance, self‐harm history, and suicidal ideation modestly attenuated but did not materially change the associations with PHQ‐9 score (adjusted *β*, −2.12; 95% CI, −2.74 to −1.50; *P* < 0.001), GAD‐7 score (adjusted *β*, −1.61; 95% CI, −2.16 to −1.06; *P* < 0.001), PHQ‐15 score (adjusted *β*, −1.36; 95% CI, −1.91 to −0.81; *P* < 0.001), functional impairment count (adjusted IRR, 0.76; 95% CI, 0.67 to 0.86; *P* < 0.001), and marked functional impairment (adjusted OR, 0.58; 95% CI, 0.43 to 0.78; *P* < 0.001). These estimates were similar to those in the main adjusted analyses, with modest attenuation in magnitude in both sensitivity analyses (Table 4).

**TABLE 4:** Sensitivity analyses of the cross‐sectional associations between BRS score and clinical burden.

Sensitivity analysis	Outcome	Model	Adjusted estimate per 1‐SD higher BRS	95% CI	*P* value
**Panel A. Exclusion of patients receiving psychotropic medication for <4 weeks (*n* = 397)**					
	PHQ‐9 score	Linear regression	*β* = −2.31	−2.96 to −1.66	<0.001
	GAD‐7 score	Linear regression	*β* = −1.76	−2.34 to −1.18	<0.001
	PHQ‐15 score	Linear regression	*β* = −1.43	−2.01 to −0.85	<0.001
	Functional impairment count	Negative binomial regression	IRR = 0.74	0.65 to 0.84	<0.001
	Moderate or greater depressive symptoms	Logistic regression	OR = 0.51	0.37 to 0.70	<0.001
	Moderate or greater anxiety symptoms	Logistic regression	OR = 0.60	0.45 to 0.81	0.001
	Moderate or greater somatic symptom burden	Logistic regression	OR = 0.65	0.48 to 0.89	0.007
	Marked functional impairment	Logistic regression	OR = 0.56	0.41 to 0.76	<0.001
**Panel B. Additional adjustment for sleep disturbance, self‐harm history, and suicidal ideation (*n* = 447)**					
	PHQ‐9 score	Linear regression	*β* = −2.12	−2.74 to −1.50	<0.001
	GAD‐7 score	Linear regression	*β* = −1.61	−2.16 to −1.06	<0.001
	PHQ‐15 score	Linear regression	*β* = −1.36	−1.91 to −0.81	<0.001
	Functional impairment count	Negative binomial regression	IRR = 0.76	0.67 to 0.86	<0.001
	Moderate or greater depressive symptoms	Logistic regression	OR = 0.54	0.39 to 0.73	<0.001
	Moderate or greater anxiety symptoms	Logistic regression	OR = 0.61	0.46 to 0.81	0.001
	Moderate or greater somatic symptom burden	Logistic regression	OR = 0.65	0.48 to 0.88	0.005
	Marked functional impairment	Logistic regression	OR = 0.58	0.43 to 0.78	<0.001

*Note*: BRS was standardized before model fitting. Panel A excluded 50 patients receiving psychotropic medication for <4 weeks, leaving 397 patients; medication status in this analysis was no current medication or current medication for ≥4 weeks. Panel B retained the full cohort and additionally adjusted the primary models for sleep disturbance, self‐harm history, and suicidal ideation during the current episode. These analyses assess robustness to treatment exposure and selected clinically relevant risk characteristics; they do not establish temporal or causal effects. Functional impairment variables remain pragmatic markers of functional burden.

Abbreviations: BRS = Brief Resilience Scale, CI = confidence interval, GAD‐7 = 7‐item Generalized Anxiety Disorder scale, IRR = incidence rate ratio, OR = odds ratio, PHQ‐9 = 9‐item Patient Health Questionnaire, PHQ‐15 = 15‐item Patient Health Questionnaire.

## 4 Discussion

In this single‐center real‐world cross‐sectional study, BRS score was concurrently associated with multiple dimensions of clinical burden among adolescents receiving psychological or psychiatric outpatient care. Lower BRS scores were associated with greater depressive, anxiety, and somatic symptom burden and with more frequent pragmatic indicators of functional burden. Descriptive and multivariable‐adjusted analyses showed the same overall inverse pattern, while the sensitivity analyses yielded similar estimates and the exploratory spline analyses were used only to examine association shape. These findings indicate that BRS score was a cross‐sectional correlate of clinical burden across several symptom and functional domains rather than being associated with only one symptom domain.

Depressive, anxiety, and somatic symptoms commonly overlap in adolescent clinical presentations and may therefore be informative when considered jointly (Campo 2012; Bohman et al. 2012; Lallukka et al. 2019). Adolescents in the outpatient setting of general hospitals usually do not exhibit only a single clear symptom of a mental disorder. Low mood, anxiety, poor sleep, fatigue, somatic complaints, school avoidance, and family difficulties may coexist within the same clinical presentation. Diagnostic categories are still important for treatment planning, but they may not fully reflect the symptom burden experienced by patients and their families (Lallukka et al. 2019; Leonhart et al. 2018). Accordingly, the present findings are best interpreted within a dimensional clinical framework (Leonhart et al. 2018; Kroenke et al. 2010). Adolescents with lower BRS scores had higher PHQ‐9, GAD‐7, and PHQ‐15 scores, indicating a concurrent association between the BRS‐measured recovery dimension and symptom burden across these domains. This overlap is particularly relevant in general‐hospital settings where somatic complaints may be a prominent reason for presentation (Campo 2012). Longitudinal studies in related populations suggest temporal links between somatic and emotional symptoms, but the present cross‐sectional data cannot establish the direction of these relationships. This cross‐sectional design cannot distinguish these possibilities, but the parallel gradients in PHQ‐9, GAD‐7, and PHQ‐15 scores support the descriptive value of evaluating emotional and somatic symptom burden together in this clinical setting.

In adolescent outpatient care, assessment of clinical burden may reasonably consider functional domains such as school attendance, daily activities, family functioning, social participation, and repeated healthcare use in addition to symptom scores (Sawyer et al. 2012; McGorry et al. 2024). The predefined marked functional impairment indicator was present in 71.4% of adolescents in the lowest BRS‐score quartile and 30.6% in the highest BRS‐score quartile. This difference provides a clinically interpretable description of concurrent functional burden beyond symptom scores alone. Descriptively, adolescents in lower BRS‐score quartiles more often had recorded disruptions in school attendance and family or social functioning.

These findings may have multiple explanations. Lower BRS scores may reflect a lower perceived capacity to recover from stress in the context of greater current distress, although the temporal direction of this relationship cannot be determined from these data. Conversely, greater depressive or anxiety symptom burden may itself reduce perceived resilience (Liu et al. 2025; Shilton et al. 2023). Somatic symptom burden and avoidance of school or social activities may also form part of the same clinical presentation. Shared contextual factors may additionally influence both perceived resilience and clinical burden. Such factors may include family adversity, parental separation, sleep disturbance, academic stress, trauma exposure, peer difficulties, and limited social support (Lee et al. 2018; Shu et al. 2024; Masten et al. 2021; Fritz et al. 2018). These explanations are plausible but were not directly tested in the present study.

The exploratory spline analyses suggested graded associations between BRS score and clinical outcomes within the observed BRS distribution. The spline analyses were intended to describe association shape across the observed BRS‐score distribution rather than to identify a clinically meaningful boundary (Smith et al. 2008; Campbell‐Sills and Stein 2007; Las‐Hayas et al. 2022). For depressive symptoms and marked functional impairment, evidence of nonlinearity was modest and should be interpreted as a minor deviation from linearity rather than as a clinically actionable threshold. This analysis did not establish diagnostic thresholds, treatment boundaries, or causal response curves. The present study does not establish BRS score as a diagnostic, prognostic, screening, or treatment‐selection rule. At most, BRS score may provide complementary descriptive context when considered alongside established symptom, functional, diagnostic, and safety assessments.

The use of routinely collected clinical data from a general‐hospital outpatient setting is both a strength and a source of methodological limitations. The sample reflects the actual adolescent patients encountered in outpatient psychological or mental treatment practices, including those with mixed diagnoses, comorbidities, variable disease courses, medication use, and histories of psychological treatment or counseling (Wu et al. 2023; Xiong et al. 2017). The cohort captures clinical heterogeneity typical of similar outpatient settings, but this does not imply generalizability to other services, regions, or populations (Lee et al. 2018). The use of brief and common self‐report measurement methods also enhances clinical feasibility. At the same time, psychometric robustness and measurement invariance remain important considerations when standardized self‐report instruments are applied across different populations and clinical contexts (Baroni et al. 2023). However, routinely collected clinical data are generally less standardized than data collected prospectively for research purposes. Diagnoses were based on routine ICD‐10 clinical coding rather than structured diagnostic interviews. Despite structured record review and senior adjudication of unclear cases, diagnostic misclassification remains possible. The pragmatic functional‐burden indicators were defined using clinically meaningful information available in routine outpatient records, but they were not validated functional scales developed through psychometric testing. Accordingly, marked functional impairment and the functional impairment count should be interpreted as pragmatic markers of functional burden rather than as validated psychometric functional outcomes.

Several additional limitations should be considered. Because BRS score and all study outcomes were measured at the same outpatient assessment, temporal ordering cannot be established. Reverse causation cannot be excluded: a lower perceived capacity to recover from stress may precede greater symptom burden, whereas greater symptom burden may also reduce this perceived capacity. Residual confounding may remain despite adjustment for demographic, diagnostic, clinical, treatment‐related, and selected risk‐related factors. Exclusion of records with missing or invalid core questionnaires or insufficient electronic medical record data may also have introduced selection bias. Important contextual factors such as trauma exposure, academic stress, peer relationships, and social support were not systematically available in the retrospective outpatient records and therefore could not be incorporated into the regression models. Patients who require urgent intervention or immediate hospitalization have been excluded, so these results may not be applicable to the most acute or at‐risk adolescents (McGorry et al. 2024).

The clinical implications of these findings should remain limited by the cross‐sectional design and the measurement characteristics of the available routine‐care data. When already available as part of routine assessment, BRS score may provide complementary descriptive context alongside symptom measures and functional, diagnostic, and safety information. A low BRS score should not be interpreted as a diagnostic, prognostic, predictive, or treatment‐guiding result. Rather, lower BRS scores should be interpreted only as a cross‐sectional correlate of greater symptom burden and more frequent pragmatic functional‐burden indicators in this cohort. Future multicenter longitudinal studies are needed to clarify temporal relationships and to determine whether BRS‐related assessment has independent longitudinal or clinical utility.

## 5 Conclusion

In this single‐center cross‐sectional outpatient study, higher BRS score was concurrently associated with lower depressive, anxiety, and somatic symptom burden among adolescents with depressive, anxiety, or somatic symptom‐related disorders. Higher BRS score was also associated with fewer pragmatic functional‐burden indicators and lower odds of the predefined marked functional impairment outcome. These findings describe concurrent associations and do not establish BRS score as a diagnostic, prognostic, predictive, or treatment‐guiding measure. Prospective multicenter studies are needed to clarify temporal relationships and to determine whether BRS‐related assessment has independent longitudinal or clinical utility.

## Author Contributions

**Longhuan Zhang**: conceptualization, data curation, formal analysis, funding acquisition, investigation, methodology, project administration, resources, software, supervision, validation, visualization, Writing – review and editing. **Meiying Zhang**: conceptualization, data curation, investigation, resources, supervision, visualization, writing – review and editing. **Xuejing Liu**: conceptualization, data curation, investigation, resources, supervision, writing – review and editing. **Lingcong Wu**: formal analysis, methodology, software, validation, writing – original draft. **Yang Zhang**: formal analysis, methodology, software, validation, writing – original draft. **Jing Shan**: formal analysis, methodology, visualization, writing – original draft.

## Funding

This research was funded by the Project of Hengshui Science and Technology Bureau: Research on Constructing Non‐pharmacological Therapies for Neurosis from the Perspective of Psychological Resilience (2024014044Z).

## Ethics Approval and Consent to Participate

The current study was approved by the Ethics Committee of the Harrison International Peace Hospital (approval number 2024238). Due to the retrospective design and anonymized data, written informed consent was waived by the Ethics Committee.

## Conflicts of Interest

The authors declare no conflicts of interest.

## Supporting information




**Supplementary Materials**: brb371811‐sup‐0001‐SuppMat.docx

## Data Availability

The datasets used and/or analyzed during the current study are available from the corresponding author on reasonable request.
