# Supplementary material for: BRS‐Assessed Resilience, Symptom Burden, and Functional Burden in Adolescents With Depressive, Anxiety, or Somatic Symptom‐Related Disorders: A Single‐Center Real‐World Cross‐Sectional Study
Source: Brain Behav. 2026 Oct 8;16(10):e71811. doi: 10.1002/brb3.71811 (PMC13648247; doi:10.1002/brb3.71811)
Supplement: Supplementary file 1 [file BRB3-16-e71811-s001.docx]

**Supplementary Tables**

**Supplementary Table S1. Internal consistency of the psychological measures in the analytic cohort**

| **Measure** | **Number of items** | **Score range** | **Cronbach's α** |
| --- | --- | --- | --- |
| Brief Resilience Scale | 6 | 1-5 | 0.79 |
| PHQ-9 | 9 | 0-27 | 0.87 |
| GAD-7 | 7 | 0-21 | 0.89 |
| PHQ-15 | 15 | 0-30 | 0.82 |

**Note:** Cronbach's α was calculated using item-level responses obtained at the index outpatient assessment. These coefficients describe internal consistency in the analytic cohort and are not intended as a full psychometric validation. BRS=Brief Resilience Scale; GAD-7=7-item Generalized Anxiety Disorder scale; PHQ-9=9-item Patient Health Questionnaire; PHQ-15=15-item Patient Health Questionnaire.

**Supplementary Table S2. Operational definitions and data sources of the pragmatic functional-burden indicators**

| **Functional-burden component** | **Operational definition** | **Assessment window** | **Source** |
| --- | --- | --- | --- |
| Symptom-related functional difficulty | Patient-reported current symptoms making usual schoolwork, daily activities, home life, or interpersonal functioning very difficult or extremely difficult | Current assessment | Routine outpatient psychological assessment form |
| School absence | Absence from school for ≥4 days because of the current psychological or somatic clinical problem | Previous 1 month | Patient or caregiver history documented in the electronic medical record |
| School suspension or interruption | Temporary suspension or interruption of regular schooling because of health or psychological problems | Previous 6 months | Electronic medical record and outpatient history |
| Repeated healthcare visits | ≥3 healthcare visits related to the current clinical problem | Previous 3 months | Electronic medical record and documented clinical history |
| Social or family functional decline | Clinician-recorded meaningful deterioration in usual social participation or family functioning | Current clinical episode | Clinician documentation in the electronic medical record |

**Note:** Marked functional impairment was operationally defined as the presence of at least one of the five indicators. The functional impairment count is the number of indicators present (range 0-5). These measures are predefined pragmatic markers of functional burden available in routine clinical records and are not a formally validated psychometric functional scale.

**Supplementary Table S3. Regression diagnostics for the main multivariable models**

| **Diagnostic domain** | **Assessment** | **Result** | **Interpretation** |
| --- | --- | --- | --- |
| Multicollinearity | VIF for single-df terms and adjusted GVIF for multi-level categorical terms | Adjusted values 1.08-1.76; maximum 1.76 | No evidence of important multicollinearity |
| Linear-model residual distribution | Residual and Q-Q plot inspection | No major deviation from approximate normality; mild tail deviation only | Acceptable for the symptom-score models |
| Homoscedasticity | Residual-versus-fitted plots | No material funnel-shaped pattern | No important heteroscedasticity identified |
| Influential observations | Cook's distance | Maximum Cook's distance 0.071; no observation >1.0 | No single observation materially influenced the models |
| Logistic-model adequacy | Inspection of continuous-predictor functional form and model residuals | No material violation affecting interpretation | Logistic models considered adequate |
| Poisson dispersion for functional impairment count | Pearson χ²/df | 1.48 | Evidence of overdispersion |
| Count-model specification | Prespecified response to observed overdispersion | Negative binomial regression used for the functional impairment count | Model choice aligned with the observed dispersion pattern |

**Note:** Diagnostics were applied to the prespecified models to identify material model violations rather than to optimize models post hoc. For multi-level categorical terms, GVIFs were interpreted after adjustment for degrees of freedom. The negative binomial count model was selected because the Poisson model showed overdispersion; no clinical inference is based on a dispersion test itself. GVIF=generalized variance inflation factor; VIF=variance inflation factor.

**Supplementary Table S4. Exploratory restricted cubic spline analyses of BRS score and clinical burden**

| **Outcome** | **Model** | **Reference BRS score** | **Effect at BRS 2.0 vs reference** | **Effect at BRS 4.0 vs reference** | **P for overall association** | **P for nonlinearity** |
| --- | --- | --- | --- | --- | --- | --- |
| PHQ-9 score | Linear regression | 2.8 | 2.90 (1.80 to 4.00) | -3.40 (-4.60 to -2.20) | <0.001 | 0.038 |
| GAD-7 score | Linear regression | 2.8 | 2.10 (1.20 to 3.10) | -3.00 (-4.10 to -1.90) | <0.001 | 0.086 |
| PHQ-15 score | Linear regression | 2.8 | 1.70 (0.80 to 2.70) | -2.50 (-3.60 to -1.40) | <0.001 | 0.112 |
| Marked functional impairment | Logistic regression | 2.8 | OR 2.08 (1.42 to 3.05) | OR 0.42 (0.26 to 0.68) | <0.001 | 0.044 |

**Note:** Restricted cubic spline models used continuous BRS score with three knots at the 10th, 50th, and 90th percentiles. The reference was BRS=2.8, corresponding to the overall mean BRS score. For continuous outcomes, effects are adjusted mean differences with 95% CIs relative to the reference; for marked functional impairment, effects are adjusted ORs. Models used the same prespecified adjustment set as the main analyses. These analyses are exploratory shape analyses and were not used to identify clinical thresholds, screening cutoffs, prognostic cutoffs, or treatment decision points. BRS=Brief Resilience Scale; CI=confidence interval; OR=odds ratio.
